# P-1822. Missed Opportunities for Hepatitis B Screening and Vaccination Among People Living with HIV in a Ryan White-Funded Clinic: A Quality Improvement Initiative

**DOI:** 10.1093/ofid/ofaf695.1991

**Published:** 2026-01-11

**Authors:** Mark Irwin, Thomas Moffitt, Tiffany Stivers, Nicole Leedy, Takaaki Kobayashi, Armaghan-e-Rehman Mansoor

**Affiliations:** University of Kentucky, Lexington, Kentucky; University of Kentucky - - Lexington, KY, Lexington, Kentucky; University of Kentucky, Lexington, Kentucky; University of Kentucky, Lexington, Kentucky; University of Kentucky, Lexington, Kentucky; University of Kentucky, Lexington, Kentucky

## Abstract

**Background:**

National guidelines from the Health Resources and Services Administration (HRSA) recommend comprehensive hepatitis B virus (HBV) screening and vaccination for all people living with HIV (PLWH). The Ryan White HIV/AIDS Program (RW) tracks HBV testing and vaccination as a performance metric. As part of a quality improvement initiative, we evaluated adherence to HBV screening and vaccination guidelines in a RW-funded academic HIV clinic and identified opportunities for intervention.

**Table 1:**
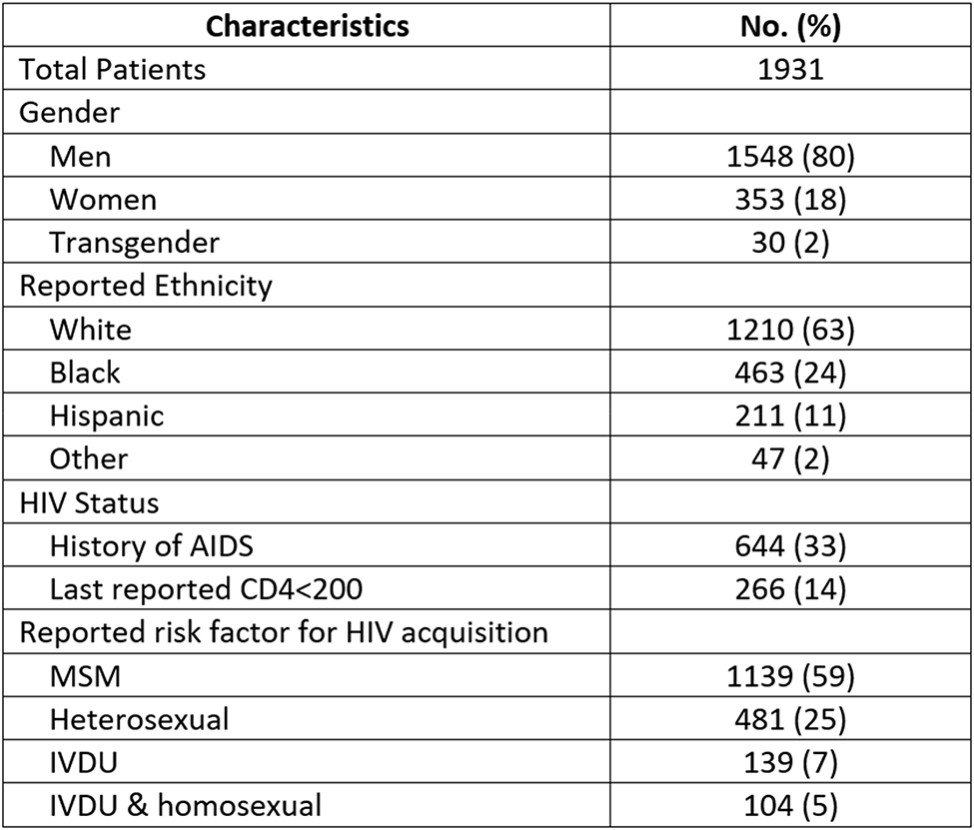
Demographics for people living with HIV in a Ryan White-funded clinic. HIV: Human Immunodeficiency Virus; AIDS: Acquired Immunodeficiency Syndrome; MSM: men who have sex with men; IVDU: intravenous drug use

**Table 2:**
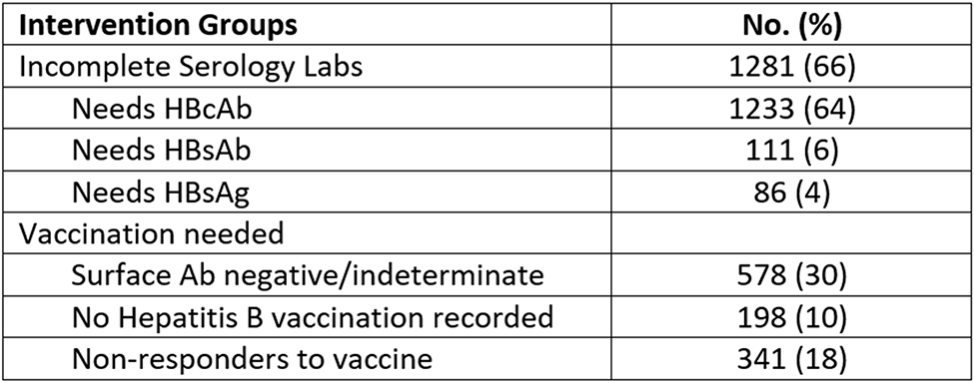
Opportunities for Hepatitis B screening and/or vaccination

**Methods:**

The CareWare database of patients enrolled at the University of Kentucky Ryan White Program was audited for data including demographics, HBV serologic testing, and vaccination status. Patients were classified into intervention groups based on completion of HBV immune status documentation, vaccination need, and documentation of post-vaccine serology in previously vaccinated PLWH.

**Results:**

A total of 1,931 patients were included. The average age was 46.8 years; 80% were male, 63% White, 24% Black, and 11% Hispanic. The most common HIV risk factor was men who have sex with men (MSM; 59%), followed by heterosexual contact (25%), injection drug use (7%), and dual risk w/ IVDU/MSM (5%). One-third (644, 33%) had a history of AIDS (CD4< 200), with 266 (14%) with a current CD4< 200.

Incomplete HBV serologies were found in 1,281 (66%) patients. The most common gap was missing HBcAb (1,233; 64%), followed by HBsAb (111; 6%) and HBsAg (86; 4%). A total of 578 (30%) had negative or indeterminate HBsAb. Among them, 198 (10%) had no prior HBV vaccination, and 341 (18%) were considered potential non-responders, having no evidence of immunity despite documented vaccination.

**Conclusion:**

In this quality improvement initiative among PLWH in a Ryan White-funded academic clinic, two-thirds had incomplete HBV serologic testing, and nearly one-third lacked confirmed immunity. We identified 539 non-mutually exclusive patients (28%) who needed HBV vaccination or evaluation for nonresponse. These findings underscore the need to incorporate HBV immunity and vaccination assessment into routine follow-up to improve compliance with national guidelines.

**Disclosures:**

All Authors: No reported disclosures

